# Injury related adult deaths in Addis Ababa, Ethiopia: analysis of data from verbal autopsy

**DOI:** 10.1186/s12889-020-08944-7

**Published:** 2020-06-15

**Authors:** Aderaw Anteneh, Bilal Shikur Endris

**Affiliations:** 1 Population Services International-Ethiopia, Addis Ababa, Ethiopia; 2 PI of AAMSP, School of Public Health, College of Health Sciences Addis Ababa University, Addis Ababa, Ethiopia

**Keywords:** Injury, Traffic accident, Deceased, Addis Ababa

## Abstract

**Background:**

Injury related deaths are causing huge impact on families and communities throughout the world. Reports show that developing countries are highly affected by injury deaths. Ethiopia is among the countries that are highly affected by injury deaths especially road traffic accident. Previous studies in Ethiopia concerning injury deaths were mostly based on hospital records. However, in the context of Ethiopia, where majority of the deaths are happening outside health facilities, hospital-based studies cannot give the exact proportion of injury deaths. This study aimed to assess the proportion and types of injury deaths in Addis Ababa and the distribution with different socio-demographic characteristics using data from verbal autopsy.

**Methods:**

We used verbal autopsy data of Addis Ababa Mortality Surveillance Program. The basic source of data for Addis Ababa Mortality Surveillance is burial surveillance from all cemeteries of Addis Ababa. We analyzed causes of injury mortality by different characteristics and tried to show the trends.

**Results:**

Over the 8 years period of time injury has contributed about 7 % of the total deaths. Majority of injury related deaths were among males. Traffic accidents were the major injury related deaths for both sexes; intentional self-harm was highly observed among males compared with females. The findings of this study showed that the proportion of injury related deaths decreased with increasing age.

**Conclusions:**

This study witnessed that deaths resulting from injuries are substantial health challenges in Addis Ababa. Road traffic accident is the most common cause of injury related deaths in the study area. The findings also indicated that males and the productive age groups are highly affected by injury deaths.

## Background

Throughout the world injury related deaths have huge impact on families and communities as majority of the deaths are attributed to the economically active age group [1]. Apart from deaths, millions of people are left with temporary or permanent disabilities as a result of injury. Each year, about 4.7 million people die as a result of injury which accounts about 9% of the world’s deaths [2]. This is more than deaths resulted from malaria, tuberculosis and Human Immunodeficiency Virus/Acquired Immune Deficiency Syndrome (HIV/AIDS) combined [2, 3]. The 2015 global burden of disease report shows that injury accounted to 10.1% of all causes of disability-adjusted life-years (DALYs) [4].

Majority of injury related deaths around the world occur in low and middle income countries [1, 5]. The 2015 World Health Organization (WHO) report shows that the highest age standardized injury related death rate (116 per 100, 000 population) occurred in Africa [6]. Ethiopia has made tremendous achievements in the health status of the people for the last three decades; life expectancy has increased from 45 in 1990 to 64.8 in 2016. The achievements are mainly due to the reductions in burden of major communicable diseases [7]. On the other hand, Ethiopia is one of the countries affected by high rates of injury related deaths especially related to road traffic accidents [8, 9]. According to the 2015 world health statistics report, the cause specific mortality due to injury in Ethiopia is 94 per 100, 000 population which is more than 8% of all deaths [6]. Ethiopia is among the countries experiencing highest road traffic accidents in the world with 79% fatality rate of injuries related with the traffic accidents [9]. Hospital visits related to injury cases account the highest number of emergency visits in many hospitals of the country [10–13]. Hospital studies in Ethiopia showed that the prevalence of injury in the emergency departments is more than 55% [11]. Road traffic accidents are the leading causes of injury related deaths among Addis Ababa residents [8, 10]. Though deaths related to road traffic accident is one of the major health challenges in Ethiopia [14], it is highly underreported [15].

The Sustainable Development Goals (SDGs) include targets for the reduction of injuries [16]. SDGs explicitly target to reduce road traffic deaths by half in 2020 [9, 16]. With the rise of non-communicable diseases and injury, the Ethiopian Health Sector Development Plan (HSDP) gives attention to injury and plans to reduce fatalities related to it [17]. In a country like Ethiopia where majority of the deaths is occurring among the economically active population [10, 18]; getting population level evidence about injury related deaths is important to achieve the plan.

Reliable data on causes of death are cornerstones for public health interventions in the allocation of resources and understanding epidemiological patterns and trends in the population [19–21]. Moreover, measuring the number of people died and the causes of death is one of the most important means for assessing the effectiveness of a country’s health system. Such information, however, are lacking in many developing countries. In a country like Ethiopia where majority of the deaths are happening outside of health facilities [22], community based verbal autopsy method is an alternative source for estimating cause specific mortality in a population. Previous studies in Ethiopia concerning injury deaths were mostly based on hospital records at certain time [8, 10–14]. However, in the Ethiopian context, hospital based studies cannot give the exact proportion of injury related deaths. This study used 8 years mortality data generated from community-based verbal autopsy. As to our knowledge, there is no similar injury related study conducted that covered all deceased in Addis Ababa. Considering high rates of injury related deaths in Addis Ababa, and lack of reliable and complete information on the contribution of injury related deaths; this paper aimed to assess the proportion and types of injury related deaths in Addis Ababa and the distribution with different socio-demographic characteristics.

## Methods

The main aim of the study is to describe the proportion of injury related deaths in Addis Ababa using verbal autopsy (VA) data. This study is conducted in Addis Ababa, Ethiopia. Addis Ababa is the capital city of Ethiopia, and it is the largest city in the country. According to the 2013 population projection of Ethiopia, the city is home to more than 3 million people of which 53% of them are females and 47% are males. The Amhara ethnic group constitute the majority of the population in the city [23].

This paper used data from Addis Ababa Mortality Surveillance Program (AAMSP). AAMSP was established in 2001 with a primary objective of monitoring the impact of HIV/AIDS on mortality in Addis Ababa [24]. It continuously collects data from all cemeteries (73) within the city limits of Addis Ababa. Cremation is not practiced in Addis Ababa; all bodies in the city are expected to be buried in formal burial sites [25]. The surveillance is conducted by cemetery based clerks who are trained about registration of basic death related data such as name of the deceased, date of burial, age, sex, place of birth, marital status, ethnicity, religion, specific address and perceived causes of death of the deceased. All cemetery clerks are assisted by supervisors to maintain completeness and consistency of data. The collected burial data were entered in a designed Microsoft Access template and cleaning is performed using a STATA do file developed for this purpose.

The data which is collected from all cemeteries of Addis Ababa serves as sampling frame for verbal autopsy (VA). Verbal autopsy is postmortem interview with next of kin or other caregivers of the deceased about the signs and symptoms during terminal illness, is an alternative method for estimating the distribution of causes of death in a population [26]. After excluding those decedents whose addresses were incomplete and bodies found dead (bodies with no close relatives or friends) a random sample of 10% of burial records were selected for VA. Considering the mourning period verbal autopsy interviews were always conducted 2 to 3 months after the death by trained interviewers.

Causes of death were ascertained by physician review. Initially two physicians reviewed the completed VA questionnaires and assigned causes of death independently. AAMSP research assistant checked the agreement between the two physician diagnoses. If the assigned cause of death for the two physicians was inconsistent it was reviewed by the third physician and final diagnosis was assigned based on the agreement between any of the two physicians. If the cause of death assigned by the third physician did not match with either of the two; the three physicians sat for panel and assigned the cause of death together. If the three physicians not agreed on the cause of death the case was labeled as undetermined. Double data entry into Microsoft Access databases were used for revised VA questionnaire and cleaning was done using STATA .do files.

Cause of death was categorized based on WHO verbal autopsy classification category [27] as injury and non-injury deaths. Further, we classified injury related deaths as road traffic accidents, intentional self-harm, assault, accidental poisoning, fall, drown, exposure to smoke and fire, legal intervention and unspecified injuries.

### Ethical considerations

The Addis Ababa Mortality surveillance program has approval from the institutional Review board of College of Health Sciences, Addis Ababa University and ethical clearance has been also guaranteed from Ethiopian Ministry of Science and Technology. The program has got authorization from the Labor and Social Affairs Bureau (the institution that manages municipal cemeteries) and all-religious authorities to conduct the surveillance at all cemeteries. Verbal Autopsy interview was conducted after obtaining written informed consent from the kin or caregiver of the deceased after explaining the purpose and procedure of the study. Respondents have given an information sheet that explains the purpose of the study and provides the contact information of the fieldwork supervisor and program manager whom they can contact for further information.

## Result

### Characteristics of the deceased

**Fig. 1:**
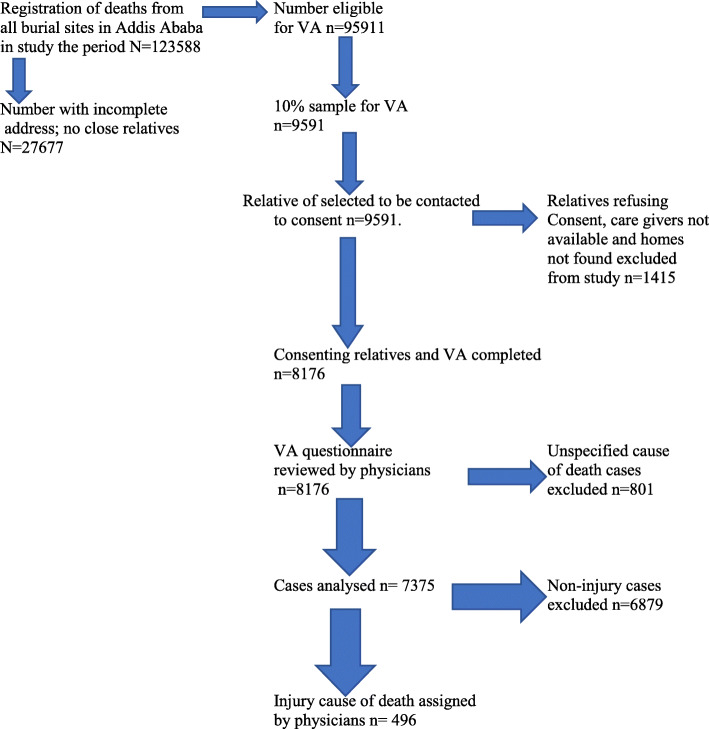
Schematic flow of Addis Ababa Mortality Surveillance Program activities

From 2007 to 2012 and 2015 to 2016, a total of 123, 588 deaths were registered at all cemeteries of Addis Ababa. Among these deaths registered, 95, 911 of them were eligible for VA sampling frame. From these eligible records, random sample of 10% (9591) were drawn for VA interview. From all the samples drawn 8, 176 were completed successfully. The remaining were not completed mainly due to family members refused to respond, care giver not available and house of the deceased were not found (Fig. 1).

The program was not functional in 2013 and 2014; hence, our analysis did not include data from 2013 and 2014. For this analysis, we excluded ill-defined causes of death (unspecified causes of death). Verbal autopsy was administered and specific cause of death was assigned for 3827 (51.9%) males and 3548 (48.1%) females during the 8 years (2007–2012& 2015–2016) study period.

Table 1 presents characteristics of deceased adults in the study period. About 33% of the deceased were in the age group 55–74 years. More than 26% of deceased were 35–54 years and about 17% of the deceased were 15–34 years. About 24% of the deceased were above 74 years of age. Concerning educational status of those deceased, 38% of them had no formal education and about a quarter of them had primary education. About 21% of deceased had secondary education and only about 10% of the deceased had above secondary education. Overall, 19% of the deceased were not married; relatively the highest proportions (44%) were married and 10% of the deceased were either divorced or separated.

**Table 1:** Socio-demographic characteristic of deceased adults (2007–2012& 2015–2016), Addis Ababa, Ethiopia

	All Deaths		Injury Deaths	
Characteristics	Number	Percent	Number	Percent
**Sex**				
Male	3827	51.9	395	79.6
Female	3548	48.1	101	20.4
**Age group in years**				
15–34	1229	16.7	218	43.9
35–54	1926	26.1	160	32.3
55–74	2428	32.9	91	18.4
Greater than 74	1792	24.3	27	5.4
**Educational Status**				
No formal Education	2842	38.5	75	15.1
Primary Education	1771	24.0	107	21.6
Secondary Education	1556	21.1	170	34.3
Above Secondary Education	701	9.5	64	12.9
Informal education	237	3.2	18	3.6
Unknown	268	3.6	62	12.5
**Religion**				
Orthodox	6500	88.1	441	88.9
Muslim	627	8.5	40	8.1
Others	248	3.4	15	3.0
**Ethnicity**				
Amhara	3823	51.8	245	49.4
Oromo	1817	24.6	127	25.6
Gurage	926	12.6	68	13.7
Others	680	9.2	55	11.1
Unknown	129	1.8	1	–
**Marital Status**				
Single	1404	19.0	229	46.2
Married	3244	44.0	229	46.2
Divorced/ Separated	733	9.9	35	7.0
Widowed	1972	26.7	2	0.4
Unknown	22	0.3	1	0.2
**Total**	**7375**	**100.0**	**496**	**100**

### Causes of injury related death

Of the verbal autopsy administered in the 8 years study period 496(6.7%) of them were injury related deaths and the remaining 93.3 were non-injury. Majority (80%) of injury related deaths affected males.

**Fig. 2:**
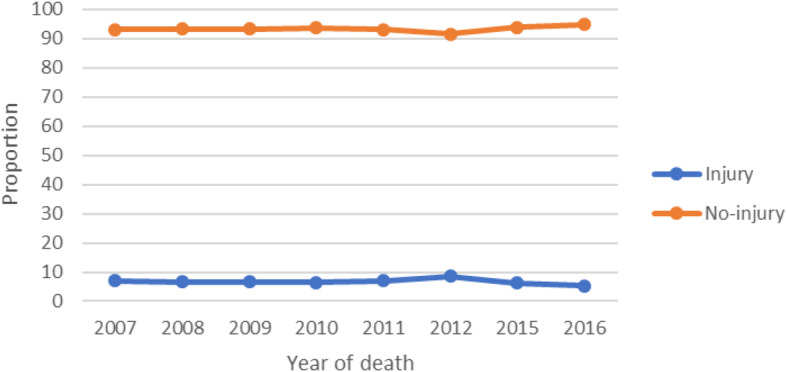
Mortality trends of causes of death among adults of Addis, Ababa, 2007-2012 and 2015-206.

Figure 2 shows the trend of causes of deaths by two groups, injury and non-injury causes of death (excluding ill-defined causes) from 2007 to 2012 and 205–2016. Over the period 2007 to 2016, the proportion of causes of death from injuries was almost similar for each year except the slight decrease in 2015 and 2016.

Table 2 shows the proportion of specific type of injury related causes of death by sex of the deceased. As indicated in the table, the greater proportion of injury deaths for both sexes were from road traffic accidents followed by intentional self-harm. The proportion of injury deaths from intentional self-harm was about 30% for males and 15% for females. Relatively higher proportions (14%) of female injury related deaths were from assault compared with 9.9% for males. Accidental fall, drown, exposure to smoke and fire together contributed about 7 and 13% of injury related deaths for male and females respectively.

**Table 2:** Different causes of injury mortality distribution by gender in adults, Addis Ababa, Ethiopia, 2007–2012& 2015–2016

Cause	Number	Percent (95% CI)		
		Male	Female	Total
Road traffic accident	198	37.9 [33.1, 42.7]	48.0 [38.2, 57.8]	39.9 [35.6, 44.2]
Intentional self-harm	132	29.6 [25.1, 34.1]	15.0 [8.0, 22.0]	26.6 [27.7, 30.5]
Assault	53	9.9 [7.0, 12.8]	14.0 [7.2, 20.8]	10.7 [8.0, 13.4]
Fall, drown, smoke and fire	47	8.6 [5.8, 11.4]	13.0 [6.4, 19.6]	9.5 [6.9, 12.1]
Accidental poisoning	18	3.8 [1.9, 5.7]	3.0	3.6 [2.0, 5.2]
Unspecified injuries	47	10.1 [7.1, 13.1]	7.0 [2.0, 12.0]	9.5 [7.0, 12.1]
**Total**	**495**	**100.00**	**100.00**	**100.00**

**Fig. 3:**
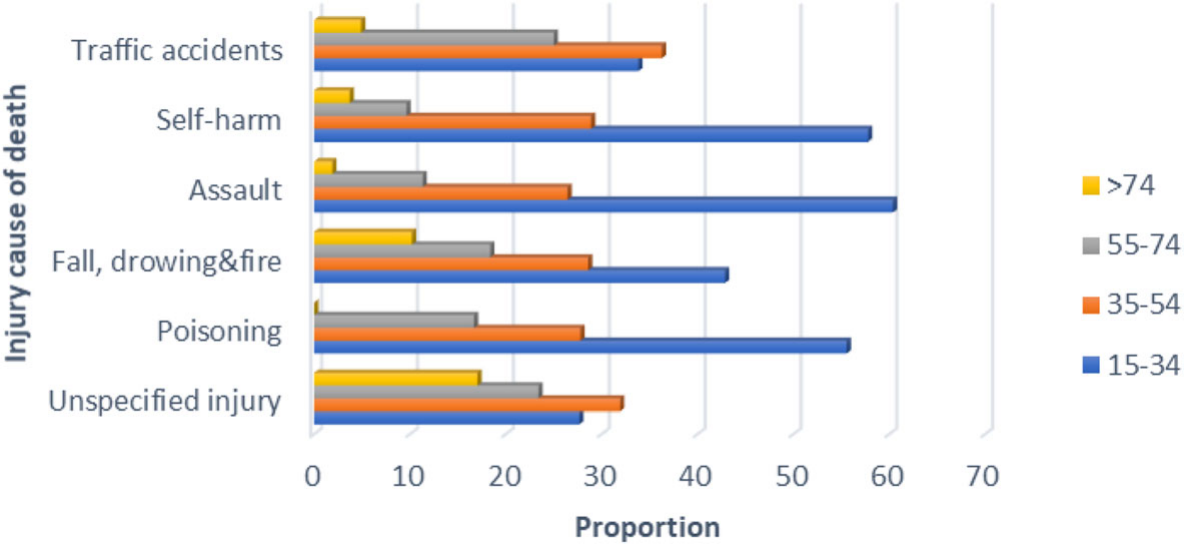
Proportion of injury causes of death for adults by age in Addis, Ababa, 2007-2016.

As depicted in Fig. 3, the proportion of all kinds of injury related deaths were greatly varied with age of the deceased. The proportion of deaths from all kinds of injury deaths highly decreased with increasing age (Fig. 3).

**Fig. 4:**
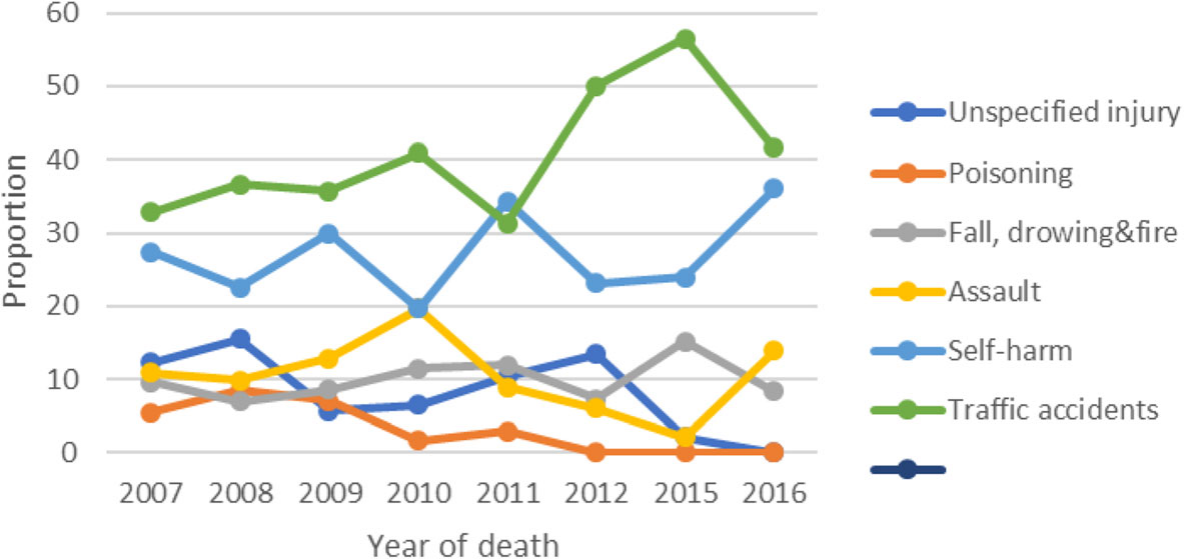
Mortality trends of the major injury of deaths among adults of Addis, Ababa, 2007-2016

Figure 4 shows the trend of injury related deaths by year of death. There has been an overall increase trend in the proportion of road traffic deaths since 2007. The result also showed that there was an increasing trend in the proportion of deaths due to assault but declined in 2011, 2012 and 2015. Deaths due to intentional self-harm has considerably varied over the years. The proportion of deaths due to poisoning has considerably decreased over the years. The proportion of deaths due to falls, drown, smoke and fire showed slight increasing trend over the years.

## Discussion

We conducted the study using data collected from all burial sites of Addis Ababa, Ethiopia supported by verbal autopsy interviews for cause of death analysis within the period of 2007 to 2012 and 2015 to 2016. In this study, we tried to show the proportion, type and distribution of injury related deaths in Addis Ababa. This study revealed that injury was attributable to 7% of all adult deaths. In addition, it indicated that majority of adult injury related deaths were males. Road traffic accident was the most common causes of injury related adult deaths, and it showed an increasing trend. The youngest age group was highly affected by injury related deaths.

We found that injury was attributable to about 7% of the total deaths. Another study in Addis Ababa using retrospective hospital data from 2001 to 2010 showed that the contribution of injury related deaths were 12% [28]. Our finding is also much lower than many hospital based studies conducted in Ethiopia where injury contributed about half of all deaths [8, 11, 12, 29]. This might be related to the difference in the design and setting of study as these studies were conducted in a hospital basis while our study used community based verbal autopsy data. Moreover, most of the studies used data from emergency departments of hospitals [8, 11, 29], where injury related visits are very high [8].

Our result showed that a large proportion of injury deaths were males. This is in line with other studies in Ethiopia that the proportion of deaths from injury is higher among men compared with women [18, 30, 31] and also in line with studies done in other African countries [32–34]. One possible explanation for the difference of male and female injury deaths is that males are highly involved in activities that can bring physical injury [32, 35, 36]. The other possibility is that males are highly involved in interpersonal violence and intentional self-harm [31–34, 37].

The most economically active people (aged 15–54) are at the greatest risk of dying as a result of injury. This finding is similar to other studies both in Ethiopia [11, 12, 28], and other developing countries [32, 33, 38]. The higher proportion of deaths from injuries for younger adults may be an indication that the younger population is highly involved to unsafe work conditions like in construction and other activities as compared with the older adults [36, 39, 40]. Majority of assault and self-harm injury deaths are attributed to the youngest age group (15–34 years). Therefore, the other possibility for the higher proportion of young adult injury deaths is that the younger adults are more subjected to violence and self-harm as compared to the older adults [32, 36].

Evidences show that road traffic accident affect the economically active population so that it brings a heavy burden on the national economy. Our finding also confirmed the growing importance of road traffic accidents in Ethiopia [10, 11, 13]. We found that road traffic accident is the leading cause of injury related deaths in Addis Ababa for both sexes. This finding is in agreement with other studies that road traffic accident is the major cause of injury related deaths in many developing countries [33, 34, 41]. This high proportion of road traffic deaths can be explained in different ways. Ethiopia is one of the least motorized countries in the world; however, there is an increase trend in the number of vehicles in the country especially in Addis Ababa [9]. Therefore, the large number of road traffic deaths may be related to the increasing motorization, poor technical quality of vehicles, higher vehicular speed, low driving skills, risky driving behavior and other high traffic law violations [14, 42–44]. Risky social behaviors like drinking of alcohol or khat chewing might also contributed for the higher proportion of road traffic deaths [44, 45]. Furthermore, the higher proportion of road traffic deaths might also be due to low awareness and vulnerability of people pathway use or low quality of pathways [43].

This study presents strong information about the contribution and type of injury related deaths in Addis Ababa. Since all deaths in Addis Ababa are considered in the sampling frame, the result can be used as a comparison for other urban areas in the country as well as other countries having similar settings. In countries where causes of death information from hospital are very limited such kind of study can give strong evidence for health planners. The result of the study can assist for health planners in the country for the reduction of injury related deaths. However, before considering the implication of the result some limitations of the study have to be considered. Exclusion of bodies that are found dead (bodies with no close relatives or friends) and incomplete addresses from the sampling frame could affect our findings. Selection biases could have been existed as some nonresidents may be buried in Addis Ababa and similarly residents can be buried outside of the city. Some of these biases were unnoticed and some of them were identified and corrected. Some of the sampled deceased were not included in the analysis; for the main reason houses of the deceased were not found and either caregivers or families of the deceased refused to give information for verbal autopsy interviews.

## Conclusion

With all the limitations stated, this is among the few studies focusing on injury deaths in Addis Ababa. Therefore, it can narrow the gaps in knowledge about deaths resulting from injuries in the country. The result of this study showed that deaths resulting from injuries are a substantial health challenge in Addis Ababa. Road traffic accident is the most common cause of injury related deaths in the study area. Our finding indicated that males and the productive age group are highly affected by injury deaths. The results of this study call the need for assessing legal frameworks related with road use and driving habits, and restriction accesses to poisons and weapons.

## Data Availability

The datasets analysed during the current study are not publicly available due to institutional policies but are available from the corresponding author on reasonable request.

## Abbreviations

HIV/AIDS: Human Immunodeficiency Virus/Acquired Immune Deficiency Syndrome
DALY: Disability-adjusted life-year
WHO: World Health Organization
SDG: Sustainable Development Goal
HSDP: Health Sector Development Plan
VA: Verbal Autopsy
AAMSP: Addis Ababa Mortality Surveillance Program
CI: Confidence Interval
